# Necessity to review the Brazilian regulation about fluoride toothpastes

**DOI:** 10.1590/S0034-8910.2015049005768

**Published:** 2015-10-05

**Authors:** Jaime Aparecido Cury, Pablo Guilherme Caldarelli, Livia Maria Andaló Tenuta

**Affiliations:** I Departamento de Ciências Fisiológicas. Faculdade de Odontologia de Piracicaba. Universidade Estadual de Campinas. Piracicaba, SP, Brasil; II Programa de Pós-Graduação em Odontologia. Faculdade de Odontologia de Piracicaba. Universidade Estadual de Campinas. Piracicaba, SP, Brasil

**Keywords:** Dentifrices, Fluoridation, legislation & jurisprudence, Brazilian Health Surveillance Agency, Dental Caries, prevention & control, Review

## Abstract

The aim of this study was to evaluate the adequacy of the Brazilian legislation about fluoride toothpaste. A search was conducted in LILACS, Medline and SciELO databases about the fluoride concentration found in Brazilians toothpastes, using descriptors on health. Publications since 1981 have shown that some Brazilian toothpastes are not able to maintain, during their expiration time, a minimum of 1,000 ppm F of soluble fluoride in the formulation. However, the Brazilian regulation (ANVISA, Resolution 79, August 28, 2000) only sets the maximum total fluoride (0.15%; 1,500 ppm F) that a toothpaste may contain but not the minimum concentration of soluble fluoride that it should contain to have anticaries potential, which according to systematic reviews should be 1,000 ppm F. Therefore, the Brazilian regulation on fluoride toothpastes needs to be revised to assure the efficacy of those products for caries control.

## INTRODUCTION

Toothbrushing with fluoride toothpastes has been pointed out as one of the factors responsible for the decline in dental caries in high^3^ and medium-income countries, such as Brazil.^10^ Besides that, the use of toothpastes may be considered the most rational method of fluoride use, because at same time that brushing promotes dental biofilm (dental plaque) disorganization, fluoride concentration is increased in the oral cavity.^12^ Nonetheless, fluoride must be chemically compatible in the toothpaste formulation to ensure anti-caries efficacy.^30^

Systematic reviews concluded that toothpaste formulations must contain concentrations of at least 1,000 ppm fluoride to provide an anti-caries effect.^21^ ^,^ ^31^ Moreover, to be able to interfere with the caries process, reducing demineralization and enhancing dental remineralization, fluoride must be chemically soluble in the formulation.^12^ Therefore, fluoride concentration and stability has been studied since the early 1980s. Those analyses have confirmed that not all toothpastes are able to maintain, until their expiration dates, the minimum soluble fluoride concentration required to yield maximum anti-caries effect.^10^ ^,^ ^13^ ^,^ ^15^ ^,^ ^28^ ^,^ ^a^

The Brazilian regulation about fluoride in toothpastes has underwent several modifications, which abolished the requirement that fluoride is soluble in the formulation.^b^ ^,^ ^c^ ^,^ ^d^ ^,^ ^e^ Thus, the current regulation in Brazil (ANVISA, Regulation 79) only establishes that total fluoride (F) concentration in toothpaste must not be higher than 0.15% (1,500 ppm F).^e^ Therefore, since this legislation was published, toothpastes with less 1,000 ppm of soluble F, or having most fluoride chemically insoluble, have been found in the market.^2^ ^,^ ^25^ The same problem has been shown with fluoride toothpastes distributed by public health care services to socially vulnerable populations.^a^

Thus, we evaluated how adequate the current Brazilian regulation governing fluoride toothpastes is.

## METHODS

A bibliographical survey was conducted, regarding the period between 1981 and 2014, in the following electronic databases: LILACS, Medline, and SciELO. The following keywords were used: Dentifrices, Toothpastes, Fluoride, and Brazil, in the Portuguese and English languages.

Papers were selected based on the following criteria: peer-reviewed studies (descriptive, observational, intervention, and literature reviews), that had been fully published in scientific journals. Titles and abstracts were used for initial selection, according to the previously defined criteria.

A form was drafted to collect the following information from fully read, selected articles: title of the article, title of the periodical, year of publication, study country, category of the study (original, review, and others), theoretical reference, method of analysis, objective, and results found.

Official documents from the Brazilian Ministry of Health were also referred to, as well as the Brazilian and international regulations on fluoride toothpastes.

## RESULTS ANALYSIS

### Dental caries decline and the importance of fluoride toothpastes

Tooth decay is a cumulative disease that affects individuals of all ages and socioeconomic levels at different extents. It is more prevalent in economically and educationally vulnerable populations.^22^ ^,^ ^23^ Even though caries indexes have significantly declined since the 1990s,^f^ ^,^ ^g^ it remains one of the biggest public health problems in Brazil.^23^ Among factors contributing to that decline, besides the decentralization in the Brazilian health care system and the fluoridation of its public water supplies, the widespread use of fluoride toothpastes stands out.^10^ ^,^ ^22^ ^,^ ^23^

In fact, the implication of fluoride toothpastes to caries decline in most high^3^ and medium-income^10^ countries has been highlighted. The reduction of dental caries, regardless of public water supply fluoridation, is known to coincide with the implementation and more widespread use of those products.^27^ Similarly, it has been shown that dental caries declined in 16 countries at the same time that fluoride was added to over 90.0% of marketed toothpastes.^24^

Particularly in Brazil, only 25.0% of toothpastes in the market were fluoridated until 1988. By then, no regulation existed in Brazil governing fluoride amounts in those products. In September 1988, fluoride was added to the top-selling toothpaste in Brazil, which had a market share of 50.0%. Thus, in 1989, the sale of fluoride toothpastes was widespread to a population scale in the country, and from there reached 90.0% of toothpaste sales.^8^ That fact represented an expressive increase in access to fluoride in public health care, as Brazil ranks third regarding *per capita* toothpaste consumption, following only the United States and Japan.^10^ Besides that increase, both in the supply and in the consumption of fluoride toothpastes, the sanitary reform that took place in Brazil led to the implementation of health care prevention programs at schools, allowing for another segment of society to also be benefited by such fluoride use.^10^ This fact may have represented a strong impact in dental caries reduction in Brazilian school-children, regardless of water fluoridation.^10^ ^,^ ^26^

The importance of fluoride toothpastes for the decline in dental caries rates that took place in Brazil has leveled off the impact of water fluoridation in the reduction of dental caries, as observed in other countries. As a matter of fact, Pereira et al^26^ (2000) followed the changes in dental caries prevalence in school-children from two municipalities, from 1991 to 1997. One of those municipalities had fluoridated water (Piracicaba, SP, Southeastern Brazil) and the other one did not (Iracemapolis, SP). In 1991, caries prevalence was 50.0% smaller in Piracicaba than in Iracemapolis. However, that percentage was gradually reduced with time, until it reached approximately 30.0% in 1997. That difference could have been even smaller if dental caries prevalence in Piracicaba was not also declining.

Nevertheless, in order that for fluoride toothpastes present anticaries potential, some minimum requirements must be met.

### Composition and stability of fluoride toothpastes

Toothpaste formulations have several components, and each of them has a specific role to ensure the desired cosmetic and preventive-therapeutic effects.^9^ However, two of them must be discussed in further depth, having in mind their significant role in the caries control-activity of fluoride toothpastes: the abrasives and fluoride as preventive-therapeutic agent.^14^ ^,^ ^20^

Regarding the preventive-therapeutic components, toothpastes should contain available fluoride (chemically-soluble fluoride) in their formulas to have anti-caries effect. In order to achieve that, the types of abrasives in formulas and the chemical forms of fluoride used must be considered.^14^ In fact, the first toothpastes that were developed in the 1960s were not fully effective in reducing dental caries, because the toothpaste was formulated with an abrasive and a fluoride salt that were chemically imcompatible.^20^

In Brazil, most toothpastes in the market are manufactured with two kinds of fluoride salts: sodium monofluorophosphate (MFP = Na_2_FPO_3_) or sodium fluoride (NaF).^28^ NaF is an inorganic salt that, in contact with water, releases fluoride as ion (F^-^). In MFP, fluoride is covalently bound to the phosphorous atom and when it is dissolved, monofluorophosphate ions (FPO_3_^2-^) are released to the solution. Fluoride as F^-^ or FPO_3_^2- ^ions released from NaF or MFP are potentially active against caries. NaF or MFP are added to toothpastes formulation depending on the abrasive systems used.^9^ ^,^ ^20^ The proper combination of fluoridated compound and abrasive systems is fundamental to ensure that the toothpaste is effective against caries.^20^

Regarding abrasive agents, they are important components in toothpastes, as they control tooth staining and help remove accumulated biofilm from the teeth during brushing. In Brazil, most toothpastes that are used by the population have calcium carbonate (CaCO_3_) as their abrasive agent.^28^ In other countries, dicalcium phosphate dihydrate (CaHPO_4_.2H_2_O) is also used as abrasive.^20^ Those toothpastes containing calcium in the abrasive have free Ca^++^ ions in their formulas, which react with ionic fluoride, forming into the toothpaste tube calcium fluoride-like (CaF_2_) insoluble salts, which do not have anticaries property.^14^

Thus, toothpastes containing Ca in their abrasives cannot be formulated with NaF, SnF_2 _or amine fluorides salts because fluoride ions present in them immediately react with Ca^++^, forming insoluble salts.^14^ ^,^ ^20^ Since insoluble fluoride salts do not have anticaries effect, silica (SiO_2_) has been used as an abrasive agent in toothpastes that are formulated with fluoride salts that produce fluoride ions. Toothpastes formulated with SiO_2 _allow that all fluoride added remain soluble throughout shelf life of the toothpaste.^14^

As fluoride ion-generating salts cannot be used in toothpaste formulations containing Ca in their abrasives, MFP was developed in the 1960s, and it has been used in toothpastes ever since.^20^ As fluoride is covalently bound to phosphate, it does not immediately react with Ca^++^ when toothpastes are produced. However, with time, MFP undergoes hydrolysis and releases fluoride ions, which react with Ca^++^ ions of the abrasive. According to the time of storage, the concentration of soluble fluoride is gradually reduced and consequently the concentration of insoluble fluoride is increased in formulation containing MFP/CaCO_3_.^29^ This fact reinforces the need for evaluating the shelf lives of toothpaste formulations and the availability of soluble fluoride in toothpastes in the market.^14^

### Fluoride concentration in Brazilian toothpastes

Both total and soluble fluoride concentrations in Brazilian toothpastes have been evaluated since the early 1980s.^6^ At that time, seven fluoride toothpastes existed in the Brazilian market and only two of them had proper soluble fluoride concentration to have anticaries potential. The evaluation of stability of Brazilian fluoride toothpastes was first conducted in the early 1980s and published in 1986.^7^ The results showed that only three of seven toothpastes analyzed had totally stable fluoride. Insoluble fluoride concentration increased with time in four toothpastes and after a year at room temperature, in one toothpaste only 20.0% of soluble fluoride was found. Thus, this study showed that Brazilian fluoride toothpastes had very different stability patterns, indicating possibly impaired anticaries effect.

In 1989, fluoride concentrations in 10 toothpastes of the Brazilian market were determined when they were purchased (fresh samples) and after accelerated aging.^8^ The results showed that only 40.0% of toothpastes had stable fluoride in their formulations. In the remaining toothpastes, insoluble fluoride percentage increased with time, ranging from 10.0% to 80.0%. These results indicated the need to regulate the quality of fluoride toothpastes in Brazil.

Ten years later, Duarte et al (1999) reported the fluoride concentration and stability in the five top-selling toothpastes in Brazil from the five regions of the country.^16^ The results showed that soluble fluoride concentrations in fresh samples were smaller than expected, decreasing by 21.0% and 44.0% after the accelerated aging process. The only toothpaste that maintained its soluble fluoride concentration with time was the one containing silica (SiO_2_) as abrasive. The authors^16^ concluded that part of fluoride was not chemically available in the most toothpastes evaluated, impairing their anticaries potential effect.

Orth et al^25^ (2001) described fluoride concentration in the five top-selling toothpastes in Brazil and in one that had been launched in the market soon after Resolution 79 from ANVISA (August 28, 2000) had become effective.^e^ Five had MFP/CaCO_3_ and one had MFP/SiO_2_ in their formulations. The data showed that in only one of the toothpastes all fluoride was soluble. In the remaining ones, insoluble fluoride percentages ranged from 6.0% to 55.0%. In the newly-launched toothpaste, total fluoride concentration was 1,423 ppm F, which complied with the recently launched regulation. However, it only had 635 ppm of soluble F to provide anticaries effect. Therefore, the authors concluded that it was mandatory to review ANVISA’s regulation to ensure population access to toothpastes containing fluoride potentially active against dental caries.

Fluoride stability was evaluated in seven toothpastes sold in Manaus, AM, under distinct environmental conditions. Five of them had MFP/CaCO_3_ and two of them had NaF/SiO_2_ in their formulations.^5^ The analyses showed that all evaluated toothpastes had soluble fluoride concentrations suitable to have anticaries potential. However, most toothpastes did not show fluoride stability after storage. In some of them, 40.0% of insoluble fluoride was found after 12 months storage at room temperature. The authors^5^ concluded that, although total fluoride concentrations in all toothpastes evaluated complied with Resolution 79 from ANVISA,^e^ the reduction of soluble fluoride concentration during storage could impair anticaries effects of some formulations.

In 2010, Cury et al^13^ analyzed 30 toothpaste brands that were being used by 206 Brazilian children from Montes Claros, MG, Southeastern Brazil. The study found that around 36.0% of MFP/CaCO_3_-based toothpastes had less than 1,000 ppm of soluble fluoride in their composition.

Ricomini et al^28^ (2012) analyzed the five top-selling fluoride toothpastes in Brazil, which were purchased in the five regions of the country. Four had MFP/CaCO_3_ and one had NaF/SiO_2_ in their formulations. The authors showed that the five evaluated toothpastes had soluble fluoride concentrations greater than 1,000 ppm F, regardless of the regions where they were purchased. Nonetheless, when fluoride concentration in those toothpastes were evaluated after they have been stored at room temperature, only one of them was able to maintain soluble fluoride concentrations above 1,000 ppm F.^15^

All previous publications reporting the problem with fluoride concentrations in toothpastes involved products that had been purchased in the Brazilian market. Conversely, Cortes et al^a^ (2012) analyzed the fluoride concentrations in two toothpastes that were distributed by public oral health care services in Sao Gabriel da Cachoeira municipality, AM, Northern Brazil, to the local indigenous population. Both had formulas with total concentrations of 1,500 ppm F. However, the average soluble fluoride concentration in one of the toothpastes was 694.7 ppm F, while in the other it was 243.9 ppm F, both below the minimum 1,000 ppm F required to ensure anticaries effect. One of those toothpastes was commercial but the other one was distributed by the *Brasil Sorridente* (Smiling Brazil) program, an initiative from the Ministry of Health.

Thus, the results found confirm the need for revising the current Resolution 79 from ANVISA,^e^ to ensure the population has access to fluoride toothpastes with anticaries effect.

### Evolution and necessity to review the Brazilian regulation on fluoride toothpastes

The first Brazilian regulation on fluoride toothpastes was established in 1989, Regulation 22 of *Secretaria Nacional de Saúde de Vigilância Sanitária *(National Health Surveillance System).^b^ That regulation established maximum and minimum soluble fluoride concentration parameters a toothpaste should have. Thus, when they were manufactured (fresh sample), toothpastes were required to have a soluble fluoride concentration of at least 1,100 ppm F and a maximum one of 1,500 ppm F. This regulation also established the minimum soluble fluoride concentrations that a toothpaste should maintain during its whole shelf life. That regulation underwent several changes, and it was superseded by later regulations from ANVISA (Table).^c^ ^,^ ^d^ ^,^ ^e^

**Table:** Evolution of the Brazilian regulation on fluoride toothpastes.

Regulation	Specifications
Regulation 22 from December 20, 1989,^b^ Secretaria Nacional de Saúde de Vigilância Sanitária (National Health Surveillance System)	Initial concentration (fresh sample) of ionic or ionizable soluble fluoride of at least 1,000 ppm and a maximum of 1,500 ppm. Fluoride compound must react with enamel or dentin. Maintenance of a minimum ionic or ionizable soluble fluoride concentration throughout the whole shelf life of the product.
Regulation 108, from September 26, 1994,^c^ ANVISA	Initial fluoride concentration (fresh sample) of at least 1,000 ppm and a maximum of 1,500 ppm. Fluoride compound must react with enamel or dentin. Maintenance of minimum fluoride concentrations throughout the whole shelf life of products.
Regulation 71 from May 29, 1996,^d^ ANVISA	Maximum concentration of 0.15%, expressed as fluoride (1,500 ppm F). Several fluoride salts can be used in toothpastes. Mixtures of allowed fluoride compounds, as long as total fluoride concentration does not exceed 0.15%.
Resolution 79, from August 28, 2000,^e^ ANVISA	Maximum concentration of 0.15%, expressed as fluoride (1,500 ppm F). Several fluoride salts can be used in toothpastes. Mixtures of allowed fluoride compounds, as long as total fluoride concentration does not exceed 0.15%.

ANVISA: *Agência Nacional de Vigilância Sanitária* (Brazilian Health Surveillance Agency)

Regulation 108 from December 26, 1994^c^ kept the same specifications as Regulation 22 concerning fluoride concentration and stability that a toothpaste should have when manufactured and throughout its shelf life. However, it did not specify that the concentration of fluoride in ppm should be soluble. Thus, this regulation would be attended even if all fluoride in a toothpaste with 1,500 ppm of total fluoride were insoluble (therefore without anticaries potential).

In 1996, a new regulation was drafted (Regulation 71 from May 29, 1996).^d^ It established that toothpastes should contain concentrations of 0.15% of fluoride (1,500 ppm F). The specification about fluoride stability was abolished but with no requirement that fluoride should be chemically soluble in the formulation. That regulation also specified that several fluoride salts could be used in toothpastes.

Resolution 79, from August 28, 2000 is currently in force in Brazil.^e^ It regulates oral and dental hygiene products. Regarding fluoride toothpaste, that resolution only determines that the maximum fluoride concentration in formulas should not exceed 0.15% (expressed as fluoride – 1,500 ppm F). It also specifies the various fluoride salts that can be used in toothpastes. Nevertheless, that regulation does not determine how much soluble fluoride (potentially active against caries) a toothpaste should contain and maintain throughout its shelf life.

The information reported above suggests that, with the evolution of Brazilian regulations on fluoride toothpastes, the main concern was safety, because the legislations only specified the maximum total fluoride amount of 0.15% that a formulation should contain. Therefore, the current regulation does not ensure that fluoride is soluble in a toothpaste to have anti-caries potential. In several publications an appeal was made to change this regulation,^5^ ^,^ ^10^ ^,^ ^25^ ^,^ ^28^ and the present publication systematically ratifies this necessity.

The problem discussed in this review is not only found in Brazil, because the legislations of other countries also do not ensure that toothpastes have minimum soluble fluoride concentration to have anticaries potential, as seen in international publications.^1^ ^,^ ^4^ ^,^ ^11^ ^,^ ^17^ ^-^ ^19^ The American legislation is an exception,^h^ because besides establishing the maximum total fluoride amount that can be added to a toothpaste, it also requires they have and maintain a minimum concentration of soluble fluoride in the formulation. On the other hand, both the European^i^ and the Mercosur legislations,^j^ similarly to Brazilian legislation, only establish the maximum amount of total fluorine that a toothpaste should contain (0.15%).

## CONCLUSIONS

Considering that:

- A fluoride toothpaste should have a concentration of at least 1,000 ppm of soluble fluoride to have anticaries effect;
- In the Brazilian market, some toothpastes containing no more than 1,500 ppm total fluoride but less than 1,000 ppm of soluble fluoride, have been found;
- The current resolution only prioritizes the safety of fluoride toothpastes, in detrimental of their anticaries effect;

ANVISA’s Regulation 79, from August 28, 2000, should be immediately revised or another legal alternative measure must be taken to ensure that none Brazilian citizen runs the risk of using a fluoride toothpaste that is ineffective against dental caries.

## Footnotes

a Cortes G, Gomes JFF, Rebelo MAB, Cury JA. Potencial anticárie dos dentifrícios distribuídos pelo Serviço Público de Saúde Bucal no Município de São Gabriel da Cachoeira, AM, Brasil. In: Anais do 10º Congresso Brasileiro de Saúde Coletiva; 14-18 nov 2012; Porto Alegre, Brasil. Brasília (DF): ABRASCO; 2013.

b Ministério da Saúde, Secretaria Nacional de Vigilância Sanitária. Portaria nº 22, de 20 de dezembro de 1989. *Diario Oficial Uniao*. 22 dez 1989; Seção II:241.

c Ministério da Saúde, Agência Nacional de Vigilância Sanitária. Portaria nº 108, de 26 de setembro de 1994. *Diario Oficial Uniao*. 28 set 1994; p.14704.

d Ministério da Saúde, Agência Nacional de Vigilância Sanitária. Portaria nº 71, de 29 de maio de 1996. Aprova a relação de documentos necessários à formação de processos para autorização, alteração e cancelamento de funcionamento de empresa, registro de produto, suas alterações, revalidação, cancelamento e outros procedimentos afins, conforme anexos I, II, III, IV, V, VI, VII, VIII, IX e X e dá outras providências. *Diario Oficial Uniao*. 4 jun 1996; p.9821-3.

e Ministério da Saúde, Agência Nacional de Vigilância Sanitária. Portaria nº 79, de 28 de agosto de 2000. *Diario Oficial Uniao.* 30 ago 2000; p.1415-1537.

f Ministério da Saúde, Secretaria de Atenção à Saúde, Departamento de Atenção Básica, Coordenação Nacional de Saúde Bucal. Projeto SB Brasil 2003: condições de saúde bucal da população brasileira 2002-2003: resultados principais. Brasília (DF); 2004 [cited 2015 Apr 30]. Available from: https://www.nescon.medicina.ufmg.br/biblioteca/imagem/1720.pdf

g Ministério da Saúde, Secretaria de Atenção à Saúde; Secretaria de Vigilância em Saúde. SB 2010: Pesquisa Nacional de Saúde Bucal: resultados principais. Brasília (DF); 2012 [cited 2015 Apr 30]. Available from: http://bvsms.saude.gov.br/bvs/publicacoes/pesquisa_nacional_saude_bucal.pdf

h US Food and Drug Administration. CFR - Code of Federal Regulations. Title 21: Food and drugs. Washington (DC); 2013 [cited 201 Apr 30]. Chapter I, Food and Drug Administration Department of Health and Human Services, Subchapter D, Drugs from human use (part 355): anticaries drugs products for over the counter human use. Available from: http://www.gpo.gov/fdsys/browse/collectionCfr.action?collectionCode=CFR&searchPath=Title+21%2FChapter+I&oldPath=Title+21&isCollapsed=true&selectedYearFrom=2013ºycord=710

i European Union. Statutory Instruments. Consumer Protection: The Cosmetic Products (Safety) Regulations 2008: nº 1284. London: Stationery Office; 2008 [cited 2015 Apr 30]. Available from: http://www.legislation.gov.uk/uksi/2008/1284/pdfs/uksi_20081284_en.pdf

j MERCOSUL. MERCOSUL/GMC/RES nº 48/02. Regulamento técnico MERCOSUL sobre lista de substâncias que os produtos de higiene pessoal, cosméticos e perfumes não devem conter, exceto nas condições e com as restrições estabelecidas**.** Brasília (DF): Sistema de Informação do Comércio Exterior; 2002 [cited 2015 Apr 30]. Available from: http://www.sice.oas.org/trade/mrcsrs/resolutions/res4802p.asp
