# Supplementary material for: Necessity to review the Brazilian regulation about fluoride toothpastes
Source: Rev Saude Publica. 2015 Oct 5;49:74. doi: 10.1590/S0034-8910.2015049005768 (PMC4603267; doi:10.1590/S0034-8910.2015049005768)
Supplement: Translation

# Translation: Necessidade de revisão da regulamentação brasileira sobre dentifrícios fluoretados

**Authors:** Jaime Aparecido Cury, Pablo Guilherme Caldarelli, Livia Maria Andaló Tenuta

**Affiliations:** I Departamento de Ciências Fisiológicas. Faculdade de Odontologia de Piracicaba. Universidade Estadual de Campinas. Piracicaba, SP, Brasil; II Programa de Pós-Graduação em Odontologia. Faculdade de Odontologia de Piracicaba. Universidade Estadual de Campinas. Piracicaba, SP, Brasil

**Keywords:** Dentifrícios, Fluoretação, legislação & jurisprudência, Agência Nacional de Vigilância Sanitária, Cárie Dentária, prevenção & controle, Revisão

## Abstract

O presente estudo analisou a adequação da regulamentação brasileira vigente sobre dentifrícios fluoretados. Foi realizada busca da literatura sobre a concentração de flúor nos dentifrícios brasileiros, tendo como fontes Lilacs, Medline e SciELO, com uso de descritores em saúde. Publicações sobre a composição de dentifrícios fluoretados brasileiros têm mostrado desde 1981 que nem todos os dentifrícios são capazes de manter durante o prazo de validade uma concentração mínima de 1.000 ppm F. Esse problema ocorre não só com dentifrícios adquiridos no mercado como com aqueles distribuídos por serviços públicos de saúde. Entretanto, a legislação brasileira (Resolução 79, Anvisa, 28/8/2000) apenas estabelece que um dentifrício não pode conter mais que 0,15% (1.500 ppm F) de flúor total, sem estabelecer o mínimo de flúor solúvel que ele deveria conter para ter potencial anticárie, a qual de acordo com revisões sistemáticas deve ser igual ou maior que 1.000 ppm F. Concluiu-se que a regulamentação brasileira sobre dentifrícios fluoretados necessita ser revisada, garantindo a eficácia desses produtos para o controle da cárie.

## INTRODUÇÃO

A escovação dental com dentifrícios fluoretados tem sido apontada como um dos fatores responsáveis pelo declínio da cárie dentária nos países de alta^3^ e média renda, como o Brasil.^10^ Além disso, a utilização de dentifrícios deve ser considerada o meio mais racional de uso de fluoreto, pois além de promover a desorganização do biofilme dental (placa bacteriana) quando somada à escovação, há aumento da concentração de flúor na cavidade bucal sempre que os dentes são escovados.^12^ No entanto, para garantir eficácia anticárie, o fluoreto deve estar presente numa formulação quimicamente compatível.^30^

Revisões sistemáticas têm concluído que as formulações de dentifrícios devem conter uma concentração de pelo menos 1.000 ppm de flúor para terem efeito anticárie.^21^ ^,^ ^31^ Além disso, essa concentração deve estar em forma quimicamente solúvel para que o fluoreto possa interferir no processo de formação da cárie, reduzindo a desmineralização e ativando a remineralização dental.^12^ Nesse sentido, a composição química em termos de concentração e estabilidade do flúor em dentifrícios tem sido estudada no Brasil desde o início da década de 1980. Essas análises têm confirmado que nem todos os dentifrícios seriam capazes de manter, até o final de seu prazo de validade, a concentração mínima de flúor solúvel necessária para o máximo efeito anticárie.^10^ ^,^ ^13^ ^,^ ^15^ ^,^ ^28^ ^,^ ^a^

Para garantir a efetividade do flúor presente nos dentifrícios brasileiros, regulamentações foram estabelecidas pela Agência Nacional de Vigilância Sanitária (Anvisa), as quais sofreram diversas modificações que culminaram na eliminação da exigência da presença de flúor solúvel em dentifrícios.^b^ ^,^ ^c^ ^,^ ^d^ ^,^ ^e^ Assim, a regulamentação vigente no Brasil, conforme Resolução Anvisa 79, de 28 de agosto de 2000, apenas estabelece que a concentração total de flúor (F) em dentifrícios não deve ser superior a 0,15% (1.500 ppm F).^e^ Dessa forma, o risco de haver no mercado um dentifrício que não contenha uma concentração de fluoreto solúvel de pelo menos 1.000 ppm F, ou que tenha a maior parte deste insolúvel (inativo contra cárie), tem sido descrito desde que a resolução entrou em vigor.^2^ ^,^ ^25^ O mesmo problema tem ocorrido com dentifrícios fluoretados distribuídos pelos serviços públicos de saúde a populações socialmente vulneráveis.^a^

O objetivo do presente estudo foi analisar a adequação da regulamentação vigente sobre dentifrícios fluoretados no Brasil.

## MÉTODOS

Foi realizado levantamento bibliográfico referente ao período de 1981 a 2014 nas seguintes bases de dados eletrônicas: Lilacs, Medline e SciELO. Foram utilizados os descritores em ciências da saúde Dentifrícios, Cremes Dentais, Flúor e Brasil, nos idiomas português e inglês.

Para seleção dos artigos foram adotados os seguintes critérios: estudos (descritivos, observacionais, intervencionais e revisões da literatura) publicados integralmente em periódicos revistos por pares. Foram inicialmente selecionados os títulos e resumos. de acordo com os critérios previamente definidos.

Para os artigos selecionados e lidos na íntegra, foi elaborado um instrumento para coletar informações referentes a: título do artigo, título do periódico, ano de publicação, país do estudo, categoria (original, revisão, e outros) do estudo, referencial teórico, método de análise, objetivo e resultados encontrados.

Foram também consultados documentos oficiais do Ministério da Saúde, bem como as regulamentações brasileiras e internacionais sobre dentifrícios fluoretados.

## ANÁLISE DOS RESULTADOS

### Declínio da cárie dentária e a importância dos dentifrícios fluoretados

A cárie dentária é uma doença cumulativa que atinge indivíduos de todas as faixas etárias e níveis socioeconômicos com diferentes magnitudes, sendo mais prevalente nas populações em situação de vulnerabilidade econômica e educacional.^22^ ^,^ ^23^ Embora tenha havido desde a década de 1990 significativo declínio dos índices dessa doença no Brasil,^f^ ^,^ ^g^ ela ainda continua sendo um dos grandes problemas de saúde pública no País.^23^ Entre os fatores que contribuíram para esse declínio, além da descentralização do sistema de saúde brasileiro e da fluoretação das águas de abastecimento público, destaca-se o uso abrangente de dentifrícios fluoretados.^10^ ^,^ ^22^ ^,^ ^23^

Assim, a importância da utilização dos dentifrícios fluoretados na explicação para o declínio da cárie dentária ocorrido na maioria dos países de alta^3^ e média renda^10^ tem sido enfatizada. Dados mostram que a redução da cárie dentária, ocorrida independentemente da fluoretação da água de abastecimento público, coincide com a implementação e a maior disseminação desses produtos.^27^ Da mesma forma, foi comprovado haver coincidência temporal entre o declínio da cárie dentária ocorrida em 16 países e a agregação de flúor em mais de 90,0% dos dentifrícios comercializados nesses países.^24^

Particularmente no Brasil, até o ano de 1988, somente 25,0% dos dentifrícios presentes no mercado eram fluoretados e até então não havia no País nenhum tipo de regulamentação sobre a quantidade do flúor nesses produtos. Em setembro de 1988, foi agregado flúor ao dentifrício mais vendido no país, responsável por cerca de 50,0% do mercado. Assim, em 1989 os dentifrícios fluoretados começaram a ser comercializados em escala populacional no País, passando a contribuir com cerca de 90,0% das vendas de dentifrícios.^8^ Este fato representou um aumento expressivo do acesso ao flúor em termos de saúde pública, pois o Brasil era o terceiro país em consumo *per capita* de dentifrícios, atrás apenas dos Estados Unidos e Japão.^10^ Além desse aumento, tanto da oferta quanto do consumo de dentifrícios fluoretados, a reforma sanitária ocorrida no Brasil levou à implementação de programas de prevenção para a saúde em escolas, permitindo que um outro segmento da sociedade também pudesse ser beneficiado por este meio de usar flúor.^10^ Dessa forma, este fato pode ter representado um importante impacto na redução da cárie dentária em escolares no Brasil, independentemente da fluoretação da água.^10^ ^,^ ^26^

A importância do dentifrício fluoretado para o declínio da cárie dentária ocorrido no Brasil tem diminuído o impacto da fluoretação da água na redução de cárie, como já ocorrido em outros países. Com efeito, Pereira et al^26^ (2000) acompanharam as mudanças da prevalência de cárie dentária em escolares de dois municípios de 1991 a 1997, um com água fluoretada (Piracicaba, SP) e outro sem fluoretação (Iracemápolis, SP). Em 1991, a diferença de prevalência de cárie era 50,0% menor em Piracicaba do que em Iracemápolis. No entanto, essa porcentagem foi gradativamente se reduzindo com o passar do tempo até chegar a aproximadamente 30,0% em 1997. Essa diferença poderia ter sido ainda menor, caso a prevalência de cárie no município de Piracicaba não estivesse também em declínio.

Entretanto, para que os dentifrícios fluoretados tenham potencial anticárie alguns requisitos mínimos devem ser atendidos.

### Composição e estabilidade dos dentifrícios fluoretados

Diversos são os componentes presentes nas formulações dos dentifrícios e cada um deles tem uma função específica para garantir os efeitos cosméticos e preventivos-terapêuticos desejados.^9^ Entretanto, dois deles devem ser discutidos com maior profundidade, tendo em vista o significativo papel que desempenham no modo de ação dos dentifrícios fluoretados no controle da cárie: os agentes abrasivos e os preventivos-terapêuticos, no qual encontram-se os fluoretos.^14^ ^,^ ^20^

Com relação aos preventivos-terapêuticos, sabe-se, por princípio, que, para ser efetivo no controle da cárie dentária, o dentifrício deve apresentar flúor disponível em sua formulação para exercer seu efeito anticárie, ou seja, flúor quimicamente solúvel. Para tanto, é fundamental considerar o tipo de abrasivo presente na formulação e a forma química em que o flúor se encontra.^14^ A esse respeito, os primeiros dentifrícios desenvolvidos na década de 1960 não foram totalmente efetivos na redução da cárie dentária, justamente devido à incompatibilidade química do flúor com os agentes abrasivos utilizados em suas composições.^20^

No Brasil, a maior parte dos dentifrícios encontrados são formulados com dois tipos de flúor, o monofluorfosfato de sódio (MFP = Na_2_FPO_3_) ou o fluoreto de sódio (NaF).^28^ Este é um sal inorgânico que, em contato com água, libera o flúor na forma de íon flúor (F^-^), que é ativo no controle da cárie. O MFP dissolvido libera na formulação de dentifrício o íon monofluorfosfato (FPO_3_^2-^), no qual o flúor está ligado covalentemente ao radical fosfato. Tanto o fluoreto na forma iônica oriundo do NaF como o íon MFP proveniente do monofluorfosfato de sódio são potencialmente ativos contra cárie e, portanto, agregados aos dentifrícios, dependendo do sistema abrasivo das formulações.^9^ ^,^ ^20^ A combinação adequada do tipo de composto fluoretado e sistema abrasivo é fundamental para garantir que a formulação tenha eficácia anticárie.^20^

Com relação aos agentes abrasivos, são importantes componentes dos dentifrícios por controlarem o manchamento dental e, durante a escovação, auxiliarem na remoção do biofilme acumulado sobre os dentes. No Brasil, a maioria dos dentifrícios utilizados pela população tem como agente abrasivo o carbonato de cálcio (CaCO_3_).^28^ Em outros países, o fosfato de cálcio di-hidratado (CaHPO_4_.2H_2_O) é usado também como abrasivo.^20^ Nesses dentifrícios contendo sais de cálcio como abrasivo, há íons Ca^++^ livres na formulação, os quais reagem com o íon flúor formando dentro do dentifrício sais insolúveis do tipo fluoreto de cálcio (CaF_2_), que não possuem atividade anticárie.^14^

Assim, dentifrícios contendo Ca no abrasivo não podem ser formulados com compostos fluoretados tipo NaF, SnF_2_ ou fluoretos de amina, pois geram íon flúor quando dissolvidos e agregados à formulação.^14^ ^,^ ^20^ O íon flúor reage imediatamente com íons Ca^++^ do abrasivo formando dentro do tubo ou bisnaga do dentifrício o CaF_2_. Este, insolúvel, não terá efeito anticárie durante a escovação dental. Dessa forma, sílica (SiO_2_) tem sido usada como agente abrasivo em dentifrícios que geram íon flúor, permitindo que todo o flúor agregado permaneça solúvel na formulação pelo prazo de sua validade.^14^

Como compostos químicos que geram íon flúor não podem ser usados em formulações de dentifrícios contendo Ca no abrasivo, o MFP foi desenvolvido na década de 1960 e tem sido até hoje utilizado em dentifrícios.^20^ Como o flúor está ligado covalentemente ao fosfato, ele não reage imediatamente com Ca^++^ quando o dentifrício é produzido. Entretanto, em função do tempo, o MFP sofre hidrólise liberando íon flúor, o qual é inativado pelos íons Ca^++^ do abrasivo. Entretanto, essa reação é lenta e com o passar do tempo há redução gradativa de flúor solúvel e consequente aumento de flúor insolúvel em formulações contendo MFP/CaCO_3_.^29^ Com isso, parte do flúor é inativado, o que reforça a necessidade de se avaliar a longevidade das formulações e a disponibilidade do flúor solúvel nos dentifrícios comercializados.^14^

### Concentração de fluoreto nos dentifrícios brasileiros

Tanto a concentração total de flúor como a solúvel em dentífricos brasileiros têm sido avaliadas desde o início da década de 1980.^6^ Nessa época, havia no comércio brasileiro apenas sete dentifrícios fluoretados, dos quais apenas dois apresentavam concentração de flúor solúvel ideal para terem potencial anticárie. A avaliação da estabilidade do flúor presente nos dentifrícios brasileiros foi realizada pela primeira vez no início dos anos 1980 e publicada em 1986.^7^ Os resultados apontaram que, quando da aquisição dos sete dentifrícios avaliados, apenas três apresentaram flúor totalmente estável. Nos demais, a concentração de flúor insolúvel (ligado ao abrasivo) aumentou em função do tempo e após um ano à temperatura ambiente, e em um deles foi encontrado apenas 20,0% de flúor solúvel. Assim, esse estudo mostrou que os dentifrícios brasileiros tinham padrões muito distintos de estabilidade, indicando um possível comprometimento do efeito anticárie.

Em 1989 foram publicados dados da avaliação do flúor encontrado em 10 dentifrícios comercializados no Brasil quando adquiridos e após envelhecimento acelerado.^8^ Os resultados mostraram que apenas 40,0% dos dentifrícios apresentavam flúor estável em suas formulações. Nos demais dentifrícios, a porcentagem de flúor insolúvel aumentou em função do tempo, variando de 10,0% a 80,0%. Estes resultados sinalizavam a necessidade de normas e regulamentações para o controle de qualidade dos dentifrícios fluoretados no Brasil, algo que até então não ocorria no País.

Dez anos após, a concentração e a estabilidade do flúor presente nos cinco dentifrícios mais vendidos no Brasil e comprados nas cinco regiões do País, foram descritas por Duarte et al (1999).^16^ Os resultados mostraram que a concentração de flúor solúvel na maioria dos dentifrícios recém-adquiridos foi menor do que o esperado, a qual se reduziu entre 21,0% e 44,0% após o processo de envelhecimento precoce. Também foi mostrado que o único dentifrício que não teve diminuição do flúor solúvel foi o que continha sílica (SiO_2_) como abrasivo. Os autores^16^ concluíram que nem todo o flúor estava solúvel nas formulações e que, durante o armazenamento, haveria inativação do flúor pelo abrasivo, comprometendo o potencial anticárie.

Orth et al^25^ (2001) descreveram as concentrações de flúor encontradas nos cinco dentifrícios mais vendidos no Brasil e de um que havia sido lançado no início da vigência da Resolução 79 da Anvisa (28/8/2000).^e^ Destes, cinco eram formulados com MFP/CaCO_3_ e um com MFP/SiO_2_. Os dados mostraram que apenas um dos dentifrícios apresentava todo seu flúor na forma solúvel. Nos demais, a porcentagem de flúor insolúvel variou de 6,0% a 55,0%. No dentifrício recém-lançado a concentração total de flúor era 1.423 ppm F, atendendo à regulamentação vigente. No entanto, nele havia apenas 635 ppm de F solúvel para exercer efeito anticárie. Com isso, os autores concluíram que era necessário revisar a regulamentação da Anvisa a fim de garantir a população o acesso a dentifrícios contendo flúor potencialmente ativo contra a cárie dentária.

A estabilidade do flúor em condições ambientais distintas foi avaliada em sete dentifrícios. Em Manaus, AM, cinco deles continham MFP/CaCO_3_ e dois deles, NaF/SiO_2_.^5^ As análises mostraram que todos os dentifrícios avaliados tinham concentração de flúor solúvel satisfatória ao controle da cárie dentária. Entretanto, a maioria das formulações não se mostrou estável quando do armazenamento, chegando algumas delas apresentar, após 12 meses de armazenamento à temperatura ambiente, 40,0% de flúor insolúvel. Os autores^5^ concluíram que, embora a concentração total de flúor encontrada em todos os dentifrícios estivesse de acordo com a Resolução 79 da Anvisa,^e^ a perda de flúor solúvel durante o armazenamento poderia comprometer o efeito anticárie de algumas formulações.

Em 2010, Cury et al^13^ realizaram uma análise de 30 marcas de dentifrícios que estavam sendo usados por 206 crianças brasileiras do município de Montes Claros, MG. O estudo mostrou que cerca de 36,0% dos dentifrícios formulados com MFP/CaCO_3_ tinham menos de 1.000 ppm de flúor na forma solúvel.

Ricomini et al^28^ (2012) analisaram os cinco dentifrícios fluoretados mais vendidos no Brasil adquiridos nas cinco regiões do país. Destes, quatro eram formulados com MFP/CaCO_3_ e um com NaF/SiO_2_. Os autores mostraram que os cinco dentifrícios avaliados apresentaram concentrações de flúor solúvel superiores a 1.000 ppm F, independentemente da região onde foram comprados. Entretanto, quando a concentração de flúor nesses dentifrícios foi avaliada após armazenamento à temperatura ambiente, apenas um dentifrício foi capaz de manter a concentração de flúor solúvel acima de 1.000 ppm F.^15^

As publicações anteriores relatando a problemática da concentração de flúor em dentifrícios foram todas realizadas avaliando produtos adquiridos no comércio brasileiro. Diferentemente, Cortes et al^a^ (2012) analisaram a concentração de fluoreto presente em dois dentifrícios distribuídos pelos serviços públicos de saúde bucal do município de São Gabriel da Cachoeira, AM, à população indígena local. Ambos eram formulados com concentração total de 1.500 ppm F. No entanto, a média da concentração de flúor solúvel encontrada em um dos dentifrícios foi 694,7 ppm F enquanto que no outro foi 243,9 ppm F, as quais eram muito inferiores à concentração mínima necessária para garantir efeito anticárie (1.000 ppm F). Um desses dentifrícios era distribuído comercialmente e o outro era oriundo do programa Brasil Sorridente, iniciativa do Ministério da Saúde.

Dessa forma, os resultados encontrados ratificam a necessidade de revisão da atual Resolução 79 da Anvisa,^e^ garantindo à população o acesso a dentifrícios fluoretados com potencial efeito anticárie.

### Evolução e necessidade de revisão da regulamentação brasileira sobre dentifrícios fluoretados

Em 1989 foi estabelecida a primeira regulamentação brasileira sobre dentifrícios fluoretados, a Portaria 22 da Secretaria Nacional de Saúde de Vigilância Sanitária.^b^ Essa portaria estabelecia parâmetros máximos e mínimos de concentração de flúor solúvel que um dentifrício deveria apresentar. Assim, quando fabricados (amostra fresca), os dentifrícios deveriam ter uma concentração de flúor solúvel de no mínimo 1.100 ppm F e no máximo de 1.500 ppm F. Também foram estabelecidas concentrações mínimas de flúor solúvel que um dentifrício deveria manter durante o prazo de validade. Essa portaria sofreu diversas modificações e foi revogada por regulamentações posteriores da Anvisa (Tabela).^c^ ^,^ ^d^ ^,^ ^e^

**Tabela:** Evolução da regulamentação brasileira sobre dentifrícios fluoretados.

Regulamentação	Especificações
Portaria 22 de 20 de dezembro de 1989,^b^ Secretaria Nacional de Saúde de Vigilância Sanitária	Concentração inicial (amostra fresca) de flúor solúvel iônico ou ionizável de, no mínimo, 1.000 ppm e, no máximo, 1.500 ppm. Composto de flúor, contido no dentifrício, reativo com esmalte e/ou dentina. Manutenção de contrações mínimas de flúor solúvel, iônico ou ionizável, até seu prazo de validade.
Portaria 108 de 26 de setembro de 1994,^c^ Anvisa	Concentração inicial (amostra fresca) de flúor de, no mínimo 1.000 ppm e, no máximo, 1.500 ppm. Composto de flúor, contido no dentifrício, reativo com esmalte e/ou dentina. Manutenção de concentrações mínimas de flúor até seu prazo de validade.
Portaria 71 de 29 de maio de 1996,^d^ Anvisa	Concentração máxima de 0,15% expresso em flúor (1.500 ppm F). Diversidade de sais de fluoreto que podem ser utilizados em dentifrícios. Mistura entre os compostos de flúor permitidos, desde que a concentração total de flúor não exceda 0,15%.
Resolução 79, de 28 de agosto de 2000,^e^ Anvisa	Concentração máxima de 0,15% expresso em flúor (1.500 ppm F). Diversidade de sais de fluoreto que podem ser utilizados em dentifrícios. Mistura entre os compostos de flúor permitidos, desde que a concentração total de flúor não exceda 0,15%.

Anvisa: Agência Nacional de Vigilância Sanitária

A Portaria 108 de 26 de setembro de 1994^c^ manteve as mesmas especificações da Portaria 22 quanto à concentração e estabilidade do flúor que um dentifrício deveria conter quando fabricado e durante seu prazo de validade. Entretanto, ela apenas se referia à concentração de flúor em ppm sem especificar a necessidade de ele estar solúvel. Assim, se houvesse no mercado um dentifrício com no máximo 1.500 ppm de flúor total, mas totalmente na forma insolúvel (inativo contra cárie), a Portaria estaria sendo atendida.

Em 1996, uma nova portaria foi regulamentada (Portaria 71 de 29 de maio de 1996),^d^ a qual estabelecia que um dentifrício deveria conter uma concentração de no máximo 0,15,0% de flúor (1.500 ppm F). Assim, ela não especificava que este flúor deveria estar em uma forma quimicamente solúvel. Essa Portaria especificava também os vários sais de fluoreto que poderiam ser utilizados em dentifrícios.

Encontra-se vigente no Brasil a Resolução 79, de 28 de agosto de 2000,^e^ a qual regulamenta os produtos de higiene dental e bucal. Quanto aos dentifrícios fluoretados,essa resolução apenas determina que a concentração máxima total flúor presente nas formulações não deve exceder 0,15% expresso em flúor (1.500 ppm F) e também especifica os diversos sais de fluoreto que podem ser utilizados em dentifrícios. Entretanto, essa Portaria não informa quanto de flúor solúvel (potencialmente ativo contra cárie) um dentifrício deveria conter e manter pelo seu prazo de validade.

O relatado mostra que na evolução da regulamentação brasileira sobre dentifrícios fluoretados foi priorizada a questão de segurança, pois apenas é especificado o máximo de 0,15% de flúor que uma formulação deve conter. No entanto, a portaria vigente não apresenta garantia de que todo esse flúor esteja solúvel para ter potencial anticárie. Diversos apelos pela necessidade da revisão dessa portaria tem sido feitos,^5^ ^,^ ^10^ ^,^ ^25^ ^,^ ^28^ sendo ratificados de forma sistemática no presente estudo.

A problemática discutida nessa revisão não é exclusiva do Brasil, pois a legislação de grande parte dos demais países não garante que um dentifrício tenha uma concentração mínima de flúor solúvel para ter potencial anticárie, como atestam publicações internacionais.^1^ ^,^ ^4^ ^,^ ^11^ ^,^ ^17^ ^-^ ^19^ Faz exceção a legislação americana,^h^ a qual, além de estabelecer o valor máximo de flúor total que os dentifrícios devem conter, requer que eles tenham uma concentração mínima de flúor solúvel. Por outro lado, tanto a legislação europeia^i^ como a do Mercosul,^j^ à semelhança da brasileira, apenas estabelecem o máximo de flúor total que um dentifrício deve conter (0,15%).

## CONCLUSÕES

Tendo em vista que:

- Um dentifrício fluoretado deve ter concentração de no mínimo 1.000 ppm de flúor solúvel para ter efeito anticárie;
- No mercado brasileiro têm sido encontrados dentifrícios que, embora apresentem não mais que 1.500 ppm de flúor total, não possuem pelo menos 1.000 ppm de flúor solúvel;
- A resolução vigente prioriza apenas a segurança do uso de dentifrício fluoretado, sendo omissa quanto ao benefício anticárie do flúor;

Conclui-se que é necessária uma revisão da Resolução da Anvisa 79 de 28 de agosto de 2000 ou que outra alternativa legal seja tomada, garantindo que nenhum cidadão brasileiro corra o risco de consumir um dentifrício fluoretado não efetivo no controle da cárie dentária.

## Footnotes

a Cortes G, Gomes JFF, Rebelo MAB, Cury JA. Potencial anticárie dos dentifrícios distribuídos pelo Serviço Público de Saúde Bucal no Município de São Gabriel da Cachoeira, AM, Brasil. In: Anais do 10º Congresso Brasileiro de Saúde Coletiva; 14-18 nov 2012; Porto Alegre, Brasil. Brasília (DF): ABRASCO; 2013.

b Ministério da Saúde, Secretaria Nacional de Vigilância Sanitária. Portaria nº 22, de 20 de dezembro de 1989. *Diario Oficial Uniao*. 22 dez 1989; Seção II:241.

c Ministério da Saúde, Agência Nacional de Vigilância Sanitária. Portaria nº 108, de 26 de setembro de 1994. *Diario Oficial Uniao*. 28 set 1994; p.14704.

d Ministério da Saúde, Agência Nacional de Vigilância Sanitária. Portaria nº 71, de 29 de maio de 1996. Aprova a relação de documentos necessários à formação de processos para autorização, alteração e cancelamento de funcionamento de empresa, registro de produto, suas alterações, revalidação, cancelamento e outros procedimentos afins, conforme anexos I, II, III, IV, V, VI, VII, VIII, IX e X e dá outras providências. *Diario Oficial Uniao*. 4 jun 1996; p.9821-3.

e Ministério da Saúde, Agência Nacional de Vigilância Sanitária. Portaria nº 79, de 28 de agosto de 2000. *Diario Oficial Uniao.* 30 ago 2000; p.1415-1537.

f Ministério da Saúde, Secretaria de Atenção à Saúde, Departamento de Atenção Básica, Coordenação Nacional de Saúde Bucal. Projeto SB Brasil 2003: condições de saúde bucal da população brasileira 2002-2003: resultados principais. Brasília (DF); 2004 [citado 2015 abr 30]. Disponível em: https://www.nescon.medicina.ufmg.br/biblioteca/imagem/1720.pdf

g Ministério da Saúde, Secretaria de Atenção à Saúde; Secretaria de Vigilância em Saúde. SB 2010: Pesquisa Nacional de Saúde Bucal: resultados principais. Brasília (DF); 2012 [citado 2015 abr 30]. Disponível em: http://bvsms.saude.gov.br/bvs/publicacoes/pesquisa_nacional_saude_bucal.pdf

h US Food and Drug Administration. CFR - Code of Federal Regulations. Title 21: Food and drugs. Washington (DC); 2013 [citado 201 abr 30]. Chapter I, Food and Drug Administration Department of Health and Human Services, Subchapter D, Drugs from human use (part 355): anticaries drugs products for over the counter human use. Disponível em: http://www.gpo.gov/fdsys/browse/collectionCfr.action?collectionCode=CFR&searchPath=Title+21%2FChapter+I&oldPath=Title+21&isCollapsed=true&selectedYearFrom=2013ºycord=710

i European Union. Statutory Instruments. Consumer Protection: The Cosmetic Products (Safety) Regulations 2008: Nº 1284. London: Stationery Office; 2008 [citado 2015 abr 30]. Disponível em: http://www.legislation.gov.uk/uksi/2008/1284/pdfs/uksi_20081284_en.pdf

j MERCOSUL. MERCOSUL/GMC/RES Nº 48/02. Regulamento técnico MERCOSUL sobre lista de substâncias que os produtos de higiene pessoal, cosméticos e perfumes não devem conter, exceto nas condições e com as restrições estabelecidas**. **Brasília (DF): Sistema de Informação do Comércio Exterior; 2002 [citado 2015 abr 30]. Disponível em: http://www.sice.oas.org/trade/mrcsrs/resolutions/res4802p.asp
